# Advancements in research leveraging phage display technology for gastric cancer diagnosis and treatment

**DOI:** 10.3389/fmicb.2025.1632560

**Published:** 2025-09-12

**Authors:** Zhenyu Wang, Yuping Li, Jun Lin, Fuyu Deng, Yao Liu, Yuan Ji

**Affiliations:** Shenzhen Institute for Drug Control Shenzhen Testing Center of Medical Devices, Shenzhen, China

**Keywords:** phage display, gastric cancer, diagnosis, Treatment, biomarker, targeting

## Abstract

Gastric cancer persists as a major global health challenge, ranking among the leading causes of cancer-related deaths worldwide. The high mortality rate primarily stems from difficulties in early detection, often resulting in late-stage diagnosis when treatment options are limited. Phage display technology, developed in 1985, has emerged as a powerful tool in gastric cancer research, facilitating significant advances in three key areas: (1) identification of novel biomarkers for early detection, (2) screening of targeted therapeutic molecules, and (3) development of diagnostic reagents. This comprehensive review examines current applications of phage display in both diagnostic and therapeutic approaches for gastric cancer, while critically analyzing existing limitations in sensitivity, specificity, library diversity, and screening efficiency. Furthermore, we discuss the promising potential of integrating phage display with other cutting-edge technologies, proposing future research directions that could enhance its clinical utility and ultimately improve patient outcomes in gastric cancer management.

## 1 Introduction

Gastric Cancer (GC) is a global malignancy and ranks as the fifth most commonly diagnosed type of cancer worldwide. Despite its high incidence, the majority of patients are diagnosed at an advanced stage with a poor prognosis due to the lack of clear clinical indications. This results in a high mortality rate, making GC the third leading cause of cancer-related deaths. It is estimated that there are over one million new cases and more than 700,000 deaths annually, with projections indicating that these figures will rise to 1.77 million new cases and 1.27 million deaths globally by 2040 (Conti et al., 2023). Early diagnosis remains the most effective strategy for improving patient survival rates (Leung et al., 2008; Jun et al., 2017). Endoscopy is commonly used in GC screening to examine tissues suspected of precancerous lesions, with a focus on the presence of chronic Atrophic Gastritis (AG) and Intestinal Metaplasia (IM) (Yue et al., 2018). However, endoscopy is not routinely used in early GC screening due to its high cost, invasiveness, and the need for specialized equipment and personnel, limiting its widespread application in GC screening. In countries or regions with lower GC incidence, non-invasive diagnostic methods such as Computed Tomography (CT) and Magnetic Resonance Imaging (MRI) are more frequently utilized. Intratumoral and intertumoral heterogeneity are prominent features of GC, contributing to its poor prognosis (Cancer Genome Atlas Research Network, 2014; Li et al., 2022). However, histological examination alone is insufficient for effective patient stratification for individualized treatment and improvement of clinical outcomes (Körfer et al., 2021). Currently, conventional GC biomarkers (such as CEA, Ca19-9, Ca 12-5, Ca 72-4) have been shown to have low sensitivity and specificity for GC detection (Yu et al., 2016) offering limited value for early GC diagnosis. Therefore, there is an urgent need to develop novel tumor biomarkers for early GC diagnosis, combined with advanced diagnostic technologies and medications, which are crucial for identifying new therapeutic targets and improving patient outcomes (Wang et al., 2021b).

Phage-mediated interactions with cancer cells and normal cells exhibit marked discrepancies between *in vitro* and *in vivo* studies. *In vitro* experiments demonstrate that phages T4 and M13 engage in nonspecific interactions with human prostate cancer PC-3 cells, modulating integrin expression and impairing cellular migration (Sanmukh and Felisbino, 2018; Sanmukh et al., 2021b). Phage MS2 primarily enters cells via caveolin-mediated endocytosis, selectively upregulating pro-oncogenic genes such as androgen receptors, AKT, and integrins, transiently affecting viability, and this may induce long-term alterations in the signaling dependencies of cancer cells, potentially offering a novel therapeutic strategy for combination regimens with AKT/MAPK pathway inhibitors (Sanmukh et al., 2021a, 2023). In normal cells, T4 phage does not activate DNA-mediated inflammatory pathways but triggers protein phosphorylation cascades that promote cell growth and survival. Researchers speculate that mammalian cells may internalize phages as biological resources to enhance cellular proliferation and metabolic processes (Bichet et al., 2023). In normal cells, phages indeed interact with eukaryotic cellular structures and enter the cells without any hindrance. Specifically, the phage vB_SauM_JS25 is capable of infiltrating cells, killing Staphylococcus aureus within them, and thereby exerting an antibacterial effect, and it does not replicate in mammalian cells (Zhang et al., 2017; Møller-Olsen et al., 2018).

*In vivo* studies have demonstrated that phages can serve as vectors for the effective targeted delivery of genes and drugs to cancer cells following systemic administration (Hajitou, 2010). Meanwhile, due to their lack of tropism for normal tissues, phages exhibit relatively low toxicity (Paoloni et al., 2009; Trepel et al., 2009). However, prolonged phage therapy may induce the production of anti-phage antibodies in the body, thereby compromising the therapeutic efficacy (Żaczek et al., 2016; Dedrick et al., 2021). In a cancer mouse model, researchers employed phage therapy to specifically deliver therapeutic genes via the AAVP-HSVtk/GCV system, thereby inducing apoptosis in tumor cells (Hajitou et al., 2006; Kia et al., 2012). Additionally, by carrying the immunomodulatory factor AAVP-TNF-α, the phage activated apoptotic signaling pathways (Tandle et al., 2009; Chongchai et al., 2021). Moreover, it could also specifically target tumor cells that highly expressed receptors such as integrins or polysialic acid (Hajitou et al., 2006; Lehti et al., 2017).

Currently, phage therapy is primarily utilized for the treatment of infections caused by multidrug-resistant bacteria (Zalewska-Piatek, 2023). Although phage therapy has demonstrated considerable promise in cancer treatment, several critical limitations persist. First, delivery efficiency remains suboptimal, with rapid phage clearance by hepatic and reticuloendothelial systems post-systemic administration (Waehler et al., 2007; Hosseinidoust, 2017). Second, physical barriers within the tumor microenvironment (e.g., dense collagen matrices) severely impede phage penetration, necessitating co-administration of matrix-degrading enzymes (e.g., collagenase) for enhanced delivery (Yata et al., 2015). Immunogenicity-related issues encompass antibody-mediated neutralization that occurs upon frequent administration [9]. Finally, phage proteins may trigger unnecessary inflammatory responses by activating the Toll-like receptor pathway (Sweere et al., 2019).

Technical constraints further complicate clinical translation: the limited cargo capacity of AAVP vectors restricts packaging of large transgenes (e.g., CRISPR-Cas9 systems), as extended capsids compromise cloning efficiency (Smith et al., 2016; Yang Zhou et al., 2020). Tumor heterogeneity introduces additional differential expression of target receptors (e.g., integrin beta3) between cancer and normal tissues leads to inconsistent therapeutic outcomes (Hood et al., 2002; Li and Lee, 2006; Kia et al., 2012). Moreover, drug-resistant cell subpopulations within tumors may partially evade the therapeutic effects (Przystal et al., 2019). Finally, with regard to the manufacturing process, the intricate production procedures of hybrid vector systems (e.g., AAVP) (Suwan et al., 2019) pose significant quality control challenges during large-scale preparation. Collectively, these limiting factors constitute critical obstacles that must be overcome in the transition of phage therapy from laboratory research to clinical application.

In contrast to cancer phage therapy, which remains at the developmental stage, phage display technology, serving as a robust molecular display and screening platform has already demonstrated significant utility in cancer research, particularly in the fields of cancer diagnosis and therapy. A comparative analysis of the applications of phage therapy and phage display technology in cancer research is provided in Table 1. By constructing libraries and screening for ligands that bind with high specificity and affinity to cancer cells, phage display technology enables precise and targeted cancer diagnostics (Shen et al., 2023). This effectively addresses the issue of low binding efficiency resulting from the reliance on genetically engineered or chemically modified phages in phage therapy. Strategies targeting tumors can be categorized into passive targeting and active targeting. Passive targeting enhances the permeability and retention effect induced by cancer cell adaptability, enabling therapeutic agents to avoid targeting normal tissues and accumulate exclusively in tumor cells, thereby reducing toxicity to normal cells (Rahim et al., 2021). On the other hand, active targeting strategies utilize drug molecules and delivery systems such as nanoparticles to deliver antitumor compounds to specific or overexpressed tumor cell receptors (Bandyopadhyay et al., 2023). However, challenges arise in therapeutic strategies, including nonspecific toxicity, compound escape from endosomes, and targeting difficulties across different cell types. Additionally, the effectiveness of passive targeting varies greatly at different stages of cancer development (Zhang et al., 2021). Peptides have demonstrated potential in mitigating these challenges when attempting to transport drugs for cellular internalization. This strategy holds promise for improving the precision of drug targeting, minimizing off-target effects, and minimizing related adverse events (Timur and Gürsoy, 2021).

**Table 1:** Comparative analysis of phage display technology versus phage therapy for cancer treatment.

**Limitations**	**Phage therapy**	**Phage display technology**
Targeting	Requires engineering, low efficiency	Direct selection of high-affinity ligands
Immunogenicity	High (prone to immune clearance)	Low (amenable to humanization)
Delivery efficiency	Poor penetration in solid tumors	Small-molecule products show strong tissue penetration
Drug-loading flexibility	Limited	Highly adaptable
Clinical maturity	Under development, immature	Established technology with multiple approved drugs

Apart from peptides, phage display technology can also be used to screen for full-length antibodies against various antigens for disease diagnosis and treatment. However, the large molecular weight of full-length antibodies limits their ability to penetrate cells and tissues. Although humanized antibody technology has reduced immunogenicity, full-length antibodies may still elicit immune responses in some patients, thereby reducing efficacy or causing adverse effects (Wang et al., 2024), compared to phage therapy, this method demonstrates significantly reduced immune responses and side effects (Jończyk et al., 2011; Alejandra et al., 2023). Phage display technology can also be employed to screen for Nanobodies (Nbs) derived from camelid animals, which exhibit high specificity and stability and are often engineered in bivalent or trivalent forms. However, large-scale production of nanobodies faces numerous challenges, including short half-life and potential immunogenicity (Bathula et al., 2021). Furthermore, phage display technology can be used to generate antibody single-chain fragment variable (scFv). These smaller antibody fragments improved tissue penetration but are limited by short half-life, reduced stability, and the absence of fragment crystallizable (Fc)-mediated effector functions (Ahmad et al., 2012).

The search for novel diagnostic or therapeutic antibodies or peptides is a formidable task, centered on the construction of phage libraries, the selection of cancer-specific targets, and the rigorous validation of ligand-receptor affinity. This review highlights the core principles of phage display technology in the context of GC diagnosis and treatment, summarizes key research, and discusses notable outcomes from the integration of phage display with other emerging technologies.

## 2 The application of phage display technology in cancer research

### 2.1 Diversity of phage vectors

Since its inception in 1985, phage display technology has evolved through continuous technological innovation into a core platform for protein engineering and drug development. In recent years, the advancement of phage display systems has been significantly driven by the remarkable optimization of vector systems. Various phage display platforms have been developed for different biotechnological applications (Table 2), including filamentous phage M13, T7, λ, and T4 of *E. coli*. Among them, filamentous phage M13 of *E. coli* is the most widely used display system. Its key feature is M13 phage does not lyse host strains and useful for panning (Chang et al., 2023). It encapsulates a single-stranded genome that encodes five distinct capsid proteins, grouped into two categories: the major capsid protein (pVIII) and the minor capsid proteins (pVII, pIX, pVI, and pIII). Most antibodies and peptides are displayed on the phage proteins pIII and pVIII. pIII determines the infectivity of the virus particles. One key advantage of using pIII is that, when phagemids are used in combination with helper phages, pIII allows for monovalent display, which facilitates the screening of high-specificity antibodies or peptides. Additionally, pIII enables the insertion of larger protein sequences (>100 amino acids) and is more tolerant than pVIII. As the major capsid protein of the Ff phage, pVIII is primarily used to enhance detection signals when the phage-displayed antibody binds to an antigen. pVI is widely used for the display of cDNA libraries, and serves as an attractive alternative to the yeast two-hybrid method due to its high-throughput capacity for identifying interacting proteins and peptides. pVI is preferred over pVIII and pIII for the expression of cDNA libraries because proteins of interest can be fused to its C-terminus without significantly disrupting its role in phage assembly (Chang et al., 2023; Istomina et al., 2024).

**Table 2:** Basic information of different types of phages.

**Phage type**	**Genome**	**Size (kb)**	**Proteins**	**Dimension**	**Display peptide**	**Familes**	**Advantages**	**Limitations**
M13	ssDNA	6.4	11	930nm^*^6.5nm	pVIII; pIII	Inoviridae	Mature technology platform; Does not lyse host strains; Useful for panning and affinity maturation	Large inserts may impair phage assembly or infectivity; limited the host range
T4	dsDNA	169	289	120nm^*^86nm	Hoc; Soc	Myoviridae	High payload capacity; High stability	Requires *in vitro* packaging, involves laborious procedures; Technically challenging
T7	dsDNA	40	55	56nm^*^29nm	gp10A; gp10B	Podoviridae	Short lytic cycle, suitable for high-throughput screening; Lyses host strains to release a high yield of phage particles; Extremely efficient expression	Precludes iterative biopanning rounds; Smaller display capacity compared to T4
λ	dsDNA	48.5	73	60nm^*^150nm	gpV; gpD	Siphoviridae	High DNA packaging efficiency; Allows for large DNA insertions; Suitable for displaying complex proteins	The lytic life cycle prevents iterative biopanning due to host cell destruction
Qβ	ssRNA	4.2	4	28nm	CP	Leviviridae	Suitable for RNA display; featuring simple structure that facilitates genetic engineering and chemical conjugation	RNA genome stability issues; Less technologically mature compared to T7/M13
MS2	ssRNA	3.6	4	26nm	CP	Leviviridae	Suitable for RNA display; Potential applications in targeting RNA	Limited display capacity; Stability and robustness issues

T4 contains two non-essential capsid proteins, HOC and SOC, which enable this dual-site display system to present multiple copies of proteins or peptides, thereby enhancing immune responses in animals. With high loading capacity and stability, T4 is utilized in the development of immunogenic products (Ren and Black, 1998; Shivachandra et al., 2007; Tao et al., 2019). However, the procedure is complex and technically challenging.

The capsid proteins of T7 phage can display peptides of up to 50 amino acids at high copy numbers, or peptides or proteins of one thousand two hundred amino acid residues at low (0.1-1 per phage) or medium (5-15 per phage). Therefore, T7 phage is widely used for screening proteins with varying molecular weights and binding affinities. Furthermore, T7 phage exhibits high stability under extreme conditions, including elevated temperatures and low pH, additionally, the T7 phage exhibits a rapid lytic cycle, which facilitates efficient and high-throughput bio-panning (Piggott and Karuso, 2016; Yu et al., 2022).

The λ-phage, a temperate phage that infects *E. coli*, utilizes its major capsid protein gpD and major tail protein gpV as fusion partners in phage display. It has High DNA packaging efficiency and allows for large DNA insertions, Unlike gpV, gpD enables multivalent display, allowing for up to two hundred and twenty copies of gpD fusion molecules per capsid. Notably, fusing proteins to either the C- or N-terminus of gpD does not interfere with phage assembly, viability, or infectivity, and facilitates unrestricted interaction between the fusion proteins and ligands or receptors. Therefore, it is particularly well-suited for expressing complex proteins (Beghetto and Gargano, 2011; Nicastro et al., 2014; Ooi and Yeh, 2024).

### 2.2 Perform phage display experiment

**Figure 1:**
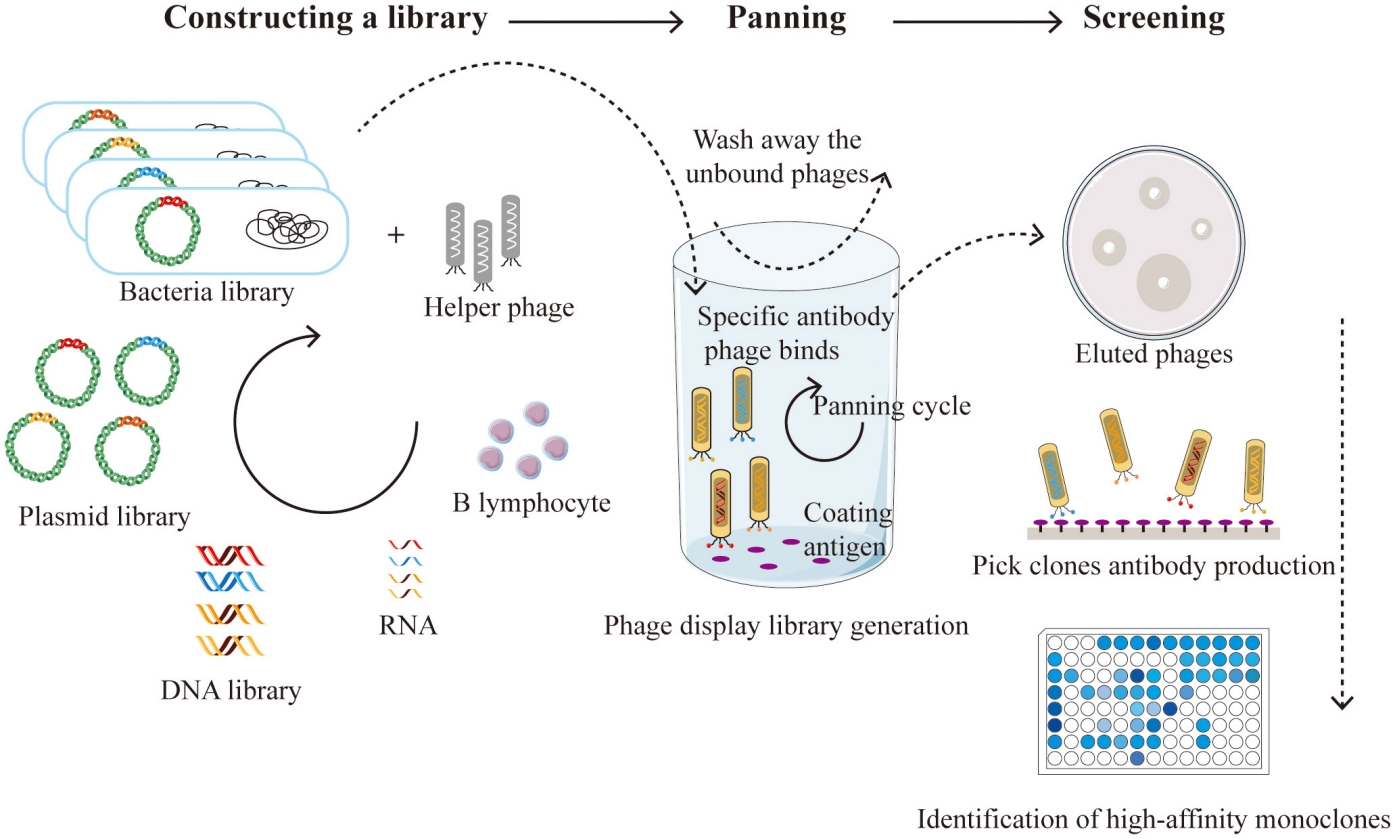
The basic steps of phage display technology are as follows. Constructing a library construction: initially, RNA is extracted from B lymphocytes and reverse transcribed into cDNA to form a cDNA library. This cDNA library is then inserted into plasmids, resulting in the creation of a microbial library. Following this, helper phages are added to construct the phage library. Panning: antigens are immobilized onto a solid support, and the phage library is applied to this surface. Phages carrying specific antibodies will bind to their corresponding antigens. Unbound phages are washed away, leaving only the bound phages on the solid support. These bound phages are then eluted and re-amplified through bacterial infection. This process is repeated several times to enrich phages for phages with higher affinity. Screening: the eluted phages are used to infect *E. coli*, and clones from these infected bacteria are selected for antibody production. Subsequently, Enzyme Linked Immunosorbent Assay (ELISA) is employed to identify monoclonal antibodies with high affinity.

The core of phage display technology lies in its highly efficient screening system, with peptide library construction being one of its key techniques. By inserting random oligonucleotide sequences into the phage genome, a library containing millions or even billions of different peptide segments can be created. This library serves as the primary source of diversity. The creation of a phage display antibody library from immune donors offers a direct method for isolating high-affinity antibodies against tumor-specific antigens using the antibody repertoire of cancer patients (Kumar et al., 2019; Zhao et al., 2023a). This process involves extracting mRNA from B lymphocytes, cloning it into the phage capsid protein gene such as pIII, and expressing these genes in *E. coli* to display the antigen-binding domains. Cancer patients often produce high-affinity antibodies due to the overexpression or mutation of tumor antigens (Ledsgaard et al., 2022). By utilizing immune phage display libraries derived from humans, researchers have established a vast antibody gene repository from cancer patients to isolate antibodies with specific binding capabilities. Taking the preparation of antibodies using M13 as an example, the basic steps of phage display are shown in the Figure 1.

## 3 The application of phage display technology in cancer research

### 3.1 The discovery of tumor markers

Utilizing phage display technology, an antibody library targeting tumor cell surface antigens can be constructed, and high-affinity, high-specificity antibodies can be screened through biopanning for use in tumor diagnosis and treatment. Based on research conducted by Philip Alexander Heine (Heine et al., 2023), genomic or metagenomic DNA is fragmented and cloned into phagemid vectors. These gene fragments are then packaged into the DNA of progeny phages via defective replicative phages, enabling the expressed protein fragments to be displayed on the surface of M13 phages. During the biopanning process, purified antibodies or serum samples are used to screen for immunogenic peptides or protein fragments that bind to the samples, with the bound fragments serving as potential biomarkers. In this approach, the phage display library is incubated with tumor cells or tumor tissues, allowing the displayed peptides or proteins to interact with surface molecules on the tumor cells. Enrichment through standard biopanning procedures results in the acquisition of highly specific binding fragments. The proteins binding to these fragments can serve as potential tumor markers. This entire process is conducted *in vitro*, requiring only DNA or cDNA from the target species. Additionally, this method is applicable to difficult-to-culture cells or metagenomic samples. Ultimately, the selected tumor markers can serve as starting points for the development of diagnostic tests, vaccines, or protein interaction studies.

### 3.2 Development of highly specific diagnostic reagents

In the process of discovering novel tumor markers using phage display technology, beyond acquiring the tumor markers themselves, DNA sequencing of the screened phages allows for the acquisition of sequence information for the displayed peptides or proteins that specifically bind to the tumor markers. This sequence information can be used to develop highly sensitive and specific diagnostic antibodies and peptide probes. These binding molecules can be applied in detection methods such as ELISA, immunohistochemistry (IHC), and biosensors, enabling precise detection of tumor markers (Saw and Song, 2019). Furthermore, phage display technology is capable of screening for binding molecules that target early-stage tumor markers or individualized markers, supporting early diagnosis and the realization of precision medicine. By incorporating the screened molecules into diagnostic reagent kits, phage display technology provides an efficient and reliable tool for cancer diagnosis, facilitating the development and application of highly specific diagnostic reagents.

### 3.3 Research on the Tumor Microenvironment (TME)

TME is a complex milieu composed of various components, including tumor-associated vasculature, the extra-cellular matrix, cancer-associated fibroblasts, tumor-associated macrophages, immune cells, and tumor cells. As the tumor progresses, these cells often undergo transformations into tumor-like phenotypes (Binnewies et al., 2018). These changes may be induced through continuous communication with other components in the TME via autocrine or paracrine mechanisms. Therefore, isolating and identifying specific peptides that target the TME can facilitate the delivery of therapeutic agents to effectively modulate or disrupt TME components through the TME homing effect. Currently, the most promising intervention sites include: (1) tumor-associated vasculature: targeting tumor angiogenesis, such as the VEGF signaling pathway, to inhibit blood vessel formation and cut off the tumor's nutrient supply (Macedo et al., 2017; Hu et al., 2021); (2) Extra-Cellular Matrix (ECM): targeting ECM components such as collagen and fibronectin to disrupt the tumor's structural support and enhance drug penetration (Lee et al., 2022; Zhang et al., 2022a); (3) Cancer-Associated Fibroblasts (CAFs): inhibiting the activation or function of CAFs to reduce their supportive role in tumor growth and metastasis (Liu et al., 2024); (4) tumor-associated macrophages (TAMs): reprogramming TAMs from a tumor-promoting phenotype (M2) to an anti-tumor phenotype (M1) to enhance the immune response (Zhao et al., 2023b).

### 3.4 Phage display technology in the preparation of antibodies

Phage display is the most widely used technology *in vitro* for antibody development. Monoclonal antibodies (mAbs) represent the most extensively applied category of recombinant drugs, playing a unique role in both diagnostic and therapeutic strategies (Ecker et al., 2015). Currently, antibody phage display libraries are employed for rapid mAb isolation. Initially, a large combinatorial library comprising variable heavy and light chain antibody libraries is constructed and expressed on the capsid protein of the phage surface, while the corresponding antibody genes are contained within the phage particles. Since the phage display is not directly influenced by *in vivo* immune responses, this strategy allows for the isolation of suitable antibodies against desired antigens from a single library in a prokaryotic system. phage display is widely used for *in vitro* antibody screening and selection. It can be applied to antibody discovery against most categories of antigens, including a broad range of epitope that may be suppressed by the immune system *in vivo* or that have never been exposed to it (Hairul Bahara et al., 2013). Antibody phage library technology enables the preparation of various antibody formats, including antigen-binding fragment (Fab), scFv, and single-domain antibody (sdAb). To date, immune phage antibody libraries established from IgG mRNA of B cells derived from immunized humans or animals have successfully screened antibodies for the treatment of various infectious diseases and cancers (Xia et al., 2006; Duan et al., 2009; Trott et al., 2014). In 2014, the FDA approved ramucirumab, developed through phage display, for the treatment of advanced GC or adenocarcinoma of esophagogastric junction, as well as in combination with docetaxel for the treatment of non-small cell lung cancer (Fuchs et al., 2014; Arrieta et al., 2017). List of phage display-derived therapeutic antibodies for gastric cancer that are either approved or have been investigated in clinical trials (Table 3).

**Table 3:** List of phage display-derived therapeutic antibodies for gastric cancer that are either approved or have been investigated in clinical trials.

**Product name**	**Target**	**Format**	**Status**	**Phage display type**	**References**
Avelumab	PD-L1	Human IgG1	Phase III	Naive Fab library	(Shim 2016); (Bang et al. 2018)
Elgemtumab	HER3	Human IgG1	Phase I	synthetic human Fab antibody library	(Malm et al. 2016); (Reynolds et al. 2017); (Takahashi et al. 2017); (Mishra et al. 2018)
Ramucirumab	VEGF Receptor 2	Human IgG1	Approved	Naive Fab library; Dyax platform	(Lu et al. 2002, 2003); (Khan and Shah 2019)
Trastuzumab	HER2	Human IgG1	Phase III	synthetic human Fab antibody library	(Bang et al. 2010); (Zhu et al. 2021)

## 4 Phage display for GC diagnostics and therapy

### 4.1 Tumor targeting peptide

Coupling therapeutic agents with target-specific phage peptides or antibodies identified through phage display allows for direct targeting of cancer cells, significantly reducing off-target effects and avoiding damage to healthy tissues. This technology enhances precision of drug delivery and significantly advances personalized diagnostics and therapy by enabling the screening of novel targeting ligands tailored to individual cancer characteristics. Targeting peptides obtained through phage display technology have been employed in the diagnosis or treatment of various types of cancers (Table 4). Researchers have utilized the Ph.D.-12 library to screen for an affinity peptide, AADNAKTKSEPV (referred to as AAD), that specifically binds to GC tissues (Zhang et al., 2012). Through three rounds of biopanning, non-specific phages binding to non-cancerous gastric mucosa were first eliminated, followed by positive selection in GC tissues, ultimately yielding the AAD peptide with high affinity. Experiments demonstrated that the AAD peptide exhibited strong binding activity in GC cells and tissues, while showing weaker binding in normal gastric mucosa and other cancer types, making it useful for distinguishing tumor tissues from normal tissues. Additionally, Dan Zhang and colleagues screened the Ph.D.-12 Phage Display Peptide Library to obtain the peptide RP-1, which targets CD44 in GC tissues, RP-1 demonstrated significantly higher fluorescence binding intensity in GC-positive tissues than in normal tissues (Zhang et al., 2015). These peptides can specifically bind to cancer tissues when labeled with fluorescence markers such as Fluorescein Isothiocyanate (FITC), providing high-contrast molecular imaging during endoscopy. When endoscopy is combined with fluorescence imaging, physicians can observe real-time fluorescent signals in cancerous areas, accurately locating early cancerous or precancerous lesions. This molecular imaging method can detect minute lesions that are difficult to identify using traditional white-light endoscopy, reducing missed diagnosis rates. Furthermore, targeted peptides can guide biopsies, ensuring that tissue samples are obtained from suspicious areas, thereby improving the accuracy and efficiency of diagnosis. In this way, molecular endoscopy combined with targeted peptides enables earlier and more precise cancer diagnosis, providing better treatment opportunities for patients.

**Table 4:** Cancer-targeting peptides developed using phage display technology.

**Cancer type**	**Target**	**Peptide/antibody sequence**	**Source**	**References**
GC	CD44	WHPWSYLWTQQA	12-mer phage peptide library	(Zhang et al. 2015)
	Nectin-4	Nbs	Immunized phage display library	(Wu et al. 2024)
	c-Met	Fab antibody	naïve Fab fragment library	(Zarei et al. 2020)
	FGFR2	scFv	Tomlinson I + J library	(Borek et al. 2018)
	GC vessels	CNTGSPYEC	Ph.D.−C7C phage display peptide library	(Zhang et al. 2024)
	LGR5	STCTRSR	Ph.D.−7 phage library	(Kwak et al. 2024)
	LGR5	IPQILSI	Ph.D.−7 phage library	(Kwak et al. 2021)
	CD44v6	ELTVMGYYPGMS	Ph.D.−12 phage display peptide library	(Zhang et al. 2020)
Breast cancer	Neuropilin-1	CLKADKAKC	Ph.D.−CX7C phage display peptide library	(Feng et al. 2014)
	Galectin-3	ANTPCGPYTHDCPVKR	f88-Cys6 library	(Zou et al. 2005)
	HER2	KCCYSL	fUSE5-cpIII phage library	(Deutscher 2010)
	DLL1	scFv	Tomlinson I + J library	(Sales-Dias et al. 2021)
	5-Lipoxygenase- Activating Protein (FLAP)	DPFYSMLQRLAH	12-mer phage-displayed library	(Jin et al. 2021)
Prostate cancer	CD44v6	PFTVSVPFVWNFTAD	fUSE5 phage library	(Peng et al. 2017)
	prostate specific antigen (PSA)^−/*lo*^	TEWDYLTV	fUSE5 phage library	(Sui et al. 2021)
	Fibroblast growth factor 8b (FGF8b)	HSQAAVP	Ph.D.−7 phage display peptide library	(Wang et al. 2013)
Lung cancer	lung cancer cell line, Calu-3	ANGRPSMT/VNGRAEAP	Landscape phage library	(Gillespie et al. 2016)
	Small Cell Lung Cancer (SCLC)and Non-Small Cell Lung Cancer (NSCLC)	GAMHLPWHMGTL/NPWEEQGYRYSM/NNPWREMMYIEI	Ph.D.−12 phage display peptide library	(Chi et al. 2017)

### 4.2 Targeted drug delivery

Conjugating drug molecules with peptides or antibodies obtained through phage display enables direct targeting of cancer cells, significantly reducing off-target effects and sparing healthy tissues from harm. This technology enhances the potential for precise drug delivery and significantly advances the development of personalized medicine by allowing the screening of novel targeting ligands tailored to individual cancer characteristics (Newman and Benoit, 2018). Yue Wu et al. successfully utilized this technology to obtain Nbs with targeted drug delivery capabilities. First, the researchers established an immune phage display library and employed phage display technology to select Nbs specific to the Nectin cell adhesion molecule 4 (Nectin-4). These Nbs were then engineered into homotrimers to enhance their affinity and subsequently fused with Nbs targeting Human Serum Albumin (HSA) to extend their *in vivo* half-life and reduce immunogenicity, resulting in trivalent humanized nanobodies. Monomethyl Auristatin E (MMAE) was site-specifically conjugated to the C-terminal site of the trivalent nanobodies, producing Nectin-4 NDC (huNb26/Nb26-Nbh-MMAE) with a drug-to-antibody ratio of 1. The Nectin-4 NDC exhibited excellent specificity and high cellular uptake in cancer cells with elevated Nectin-4 expression, and effectively inhibited GC progression *in vivo* in a dose-dependent manner (Wu et al., 2024). In another study, phage display technology was used to screen and identify a hydrophilic heptamer peptide sequence STCTRSR (referred to as STC). Compared with healthy cells, GC cells showed significantly increased uptake of a fluorescent dye, Chlorin e6 (Ce6), when conjugated to the STC peptide. Relative to free Ce6, Ce6-STC conjugates demonstrated 3.4 times higher fluorescence intensity in tumor tissues and generated greater phototoxicity following a single laser irradiation. Repeated photodynamic therapy further reduce tumor volume. The STC peptide, as a GC-specific probe, holds promise for both the diagnosis and treatment of GC. Additionally, considering its targeting ability and hydrophilicity, the STC peptide may be further explored for delivering hydrophobic chemotherapeutic drugs to GC, enhancing therapeutic efficacy (Kwak et al., 2024). In summary, studies have confirmed that targeted drugs conjugated with peptides or antibodies significantly improve drug uptake within cancer cells compared to non-targeted drugs.

### 4.3 Imaging applications for GC

Numerous studies have demonstrated that screening phage libraries has emerged as a pivotal method for identifying phage peptides or ligands that target uniquely expressed molecules on GC cells, thereby facilitating early detection and precise diagnosis. Engineered phage-displayed peptides can be conjugated with fluorescent markers or radiolabeled compounds, enabling direct visualization and mapping of GC cells and lesions. In one study, researchers targets Leucine-rich repeat-containing G-protein coupled receptor 5 (LGR5), a stem cell marker in GC, using phage display technology to produce peptide probes for molecular imaging. A novel 7-mer peptides, IPQILSI (referred to as IPQ^*^), was identified. When labeled with FITC or cyanine 5.5 (Cy5.5), the peptide exhibited a 2 to 10-fold increase in fluorescence intensity in GC cells compared to control cells. This distinction was consistently observed in immune cytochemical assays using Cy5.5-conjugated IPQ^*^. Flow cytometry (FACS) analysis revealed a rightward shift inflorescence peaks for GC cells relative to control cells. In a peritoneal metastasis animal model, Cy5.5-conjugated IPQ^*^ specifically accumulated in GC cells, suggesting that its potential as a targeted molecular imaging probe for GC detection (Kwak et al., 2021).

In a related study on colorectal cancer, phage display technology was used to select disulfide-restricted heptapeptides that bind human gastric mucin MUC5AC. These peptides were conjugated with Ultrasmall Particles of Iron Oxide (USPIOs) to form a contrast agent. The USPIOs accumulated in cancerous tissues and can be detected via Magnetic Resonance Imaging (MRI), indicating the potential for phage-derived contrast agents in non-invasive imaging of GC (Rossez et al., 2016). Recent global clinical trials have identified CLDN18.2 as an ideal target for GC treatment, with patients exhibiting high CLDN18.2 expression benefiting from targeted therapy. Researchers screened a phage display library with a capacity of 100 billion variants and identified a peptide named T37 that specifically recognizes CLDN18.2. When combined with gallium-68 (68Ga) and the chelator DOTA (1,4,7,10-tetraazacyclododecane-1,4,7,10-tetraacetic acid), the resulting probe 68Ga-DOTA-T37 exhibited good biosafety and enabled specific tomography/computed tomography (PET/CT) imaging of CLDN18.2 positive expressing tumors in mouse (Wang et al., 2023). These research cases illustrate that peptides obtained through phage display technology can be artificially modified into molecular probes compatible with various cancer-related imaging techniques.

## 5 Future challenges and directions

### 5.1 Limitations of phage display technology

In the phage display system, phage peptides are displayed through coupling with the coat proteins of filamentous phage. However, the small size of these phages limits the size of the proteins that can be displayed (Mahdavi et al., 2022). When phage display technology is used for antibody preparation *in vitro*, the antibody light chain and heavy chain are not maintained in their natural paired form. During the immune process, the Variable Heavy chain (VH) and Variable Light chain (VL) undergo paired affinity maturation. However, in cloning, VH and VL are typically amplified separately via PCR and then randomly reassembled into scFv or Fab constructs, which can result in the loss of affinity maturation advantage. Furthermore, phage are not well-suited for displaying proteins that require post-translational modifications, such as phosphorylation or glycosylation. Modifications are crucial for correct protein folding, molecular interactions, and signal transduction. This limitation restricts the broader application of phage display in study proteins that depend on these modifications (Qi et al., 2021). To address these issues, phage display can be combined with other surface display technologies, such as yeast and mammalian display platforms, which enhance capabilities for post-translational modifications due to eukaryotic system. Rare clones targeting low-immunogenicity and complex epitopes are difficult to isolate using conventional phage display methods. To obtain rare hapten-specific clones, some studies have integrated phage display with yeast display systems and competitive flow cytometry analysis, significantly increasing the proportion of hapten-specific scFv (Sun et al., 2016).

Moreover, antibodies or peptides selected via *in vitro* screening might fail to replicate the intricate physiological conditions present *in vivo*, for instance, screening using an *in vitro* blood-brain barrier (BBB) model, researchers identified FC5, a camelid antibody targeting TMEM30A that demonstrates the capacity to deliver therapeutically relevant drug payloads to brain tissue (Muruganandam et al., 2002; Abulrob et al., 2005; Farrington et al., 2014). However, although numerous cell-based *in vitro* BBB models are currently available for screening purposes, the loss of critical BBB properties under culture conditions precludes definitive assurance that the identified antibodies and their molecular targets will retain functionality *in vivo* applications (Helms et al., 2016).

### 5.2 Innovations in phage display technology: the integrated application of advanced library design and next-generation sequencing (NGS)

The traditional phage display technology faces limitations such as restricted library diversity and inefficient panning, which can be addressed by advanced library design integrated with NGS. Sophisticated library design methodologies introduce chemical or enzymatic modifications and non-natural amino acids (nnAAs) into the phage libraries, thereby enhancing the molecular diversity displayed on the phages. These innovative libraries expand the potential for identifying high-quality binders against challenging or previously inaccessible targets (Zhang, 2023; Chen et al., 2024). Furthermore, for rare clones with low representation, to obtain more comprehensive information about the sequences obtained through traditional biological panning and to minimize the loss of potentially effective sequences, NGS serves as a powerful complementary tool. Unlike traditional methods that typically yield only a few dozen sequences„ NGS allows for the collection of information from millions of sequences, reducing the risk of overlooking rare but functionally important variants lost during wash steps. For instance, when preparing scFv targeting the BBB, a high proportion of non-specific phage accumulation results in significant background noise, thereby obscuring the truly targeting antibodies. NGS technology enables high-throughput sequencing of the CDR3 sequences from all collected phages, assisting researchers in uncovering rare clones with low abundance but enrichment characteristics amidst the high background (Stutz et al., 2018). These rare clones might be overlooked by traditional methods. In addition, NGS allows for detailed examination of sequence similarity and evolutionary convergence across large datasets, offering deeper insights into binding preferences and selection dynamics (Juds et al., 2020; Andreu-Sánchez et al., 2023).

### 5.3 Microfluidics, an effective tool for supporting phage display

Moreover, microfluidic can optimize various aspects of phage display technology. By precisely controlling fluid flow, microfluidic technology facilitates effective interactions between target proteins or cells and the phage library, ensuring that a sufficient number of specific phages are enriched, thereby improving the accuracy of subsequent screening. The washing steps in traditional phage display technology typically involve multiple centrifugation and washing operations, which are both time-consuming and prone to phage loss. In contrast, microfluidic technology can effectively remove unbound phages by incorporating specifically designed microchannel structures and leveraging fluid dynamic or acoustic effects (Wang et al., 2011). Additionally, clone identification in traditional phage display technology often requires multiple ELISA validations, making the process labor-intensive and time-consuming. Microfluidic technology addresses this issue by encapsulating individual phages and target-coated beads in water-in-oil droplets, enabling single-clone phage amplification and target binding within the chip. Afterward, the droplets are broken and the beads are washed to remove most nonspecific phages. Subsequently, specific phages on the magnetic beads are stained with the fluorescently labeled M13 phage antibody, and the clones exhibiting the highest fluorescence intensity are collected and sequenced without the need for further ELISA validation of binding affinities (Wang et al., 2021a).

### 5.4 Artificial intelligence (AI) drives industrial development

In the rapid evolution of AI today, although the preparation of peptides or antibodies through phage display remains confined to the laboratory, the immense value brought by AI cannot be overlooked. The rapid advancement of NGS has generated an unprecedented amount of antibody data, providing a crucial foundation for AI-driven optimization. AI is increasingly being used to refine the physicochemical properties of antibodies or peptides, such as affinity and stability (Vascon et al., 2020; Xiang et al., 2021; Harvey et al., 2022). Furthermore, it can guide rational antibody modeling and structural optimization, simulate ligand binding, and predict binding sites, all while maximizing time and cost efficiency. The extensive learning capabilities of deep neural networks allows them to autonomously extract multifaceted features from a wide array of data types, supporting the development of highly adaptable and robust models. Notably, cutting-edge architectures such as Graph Neural Networks (GNNs) and Transformers, have exhibited remarkable performance (LeCun et al., 2015; Zhang et al., 2022b). In the realm of biomedicine, AI can accurately detect and predict disease-associated genetic variations and clinical outcomes by analyzing extensive genomic or imaging datasets (Esteva et al., 2017; Poplin et al., 2018; Wainberg et al., 2018; Zou et al., 2019). It is heartening to note that publicly available antibody-related databases are rapidly improving, serving as a solid cornerstone for providing higher-quality training data to deep learning models. Based on these data, more accurate AI models can be trained, further enhancing predictive accuracy (Vascon et al., 2020). However, these technologies can be challenging to understand for researchers without a background in computer programming. As AI technology continues to advance, the development of intuitive and user-friendly tools will be crucial for empowering non-programmers to harness the full potential of AI in biomedical research.

## 6 Conclusion

GC is a significant contributor to mortality worldwide, yet the availability of specific and high-affinity agents for early detection remains limited. Phage display technology provides robust support for early diagnosis during the asymptomatic phase of GC, as well as for targeted drug delivery and cancer imaging. By screening and identifying phage peptides with high binding affinity to GC cells, phage display facilitates rapid isolation and recognition of these cells, thereby enhancing the speed and efficiency of immunodiagnostics. Phage display serves as a powerful tool for drug discovery and biomarker imaging and has proven its value as a reliable platform for pharmaceutical development. Several anticancer drugs derived from phage display, including Ramucirumab, Necitumumab, and Avelumab, have been approved by the FDA (Casak et al., 2015; Garnock-Jones, 2016; Lohray et al., 2023). This article higlights both the importance and limitations of ongoing research utilizing phage display technology. With continued optimization of technical processes and integration with emerging technologies, phage display holds great promise for playing an even more pivotal role in future cancer research and clinical practice, offering increasingly precise and effective diagnostic and therapeutic solutions for GC patients.

## References

[B1] Abulrob A. Sprong H. Van Bergen en Henegouwen P. Stanimirovic D. (2005). The blood-brain barrier transmigrating single domain antibody: mechanisms of transport and antigenic epitopes in human brain endothelial cells. J Neurochem. 95, 1201–1214. 10.1111/j.1471-4159.2005.03463.x 16271053

[B2] Ahmad Z. A. Yeap S. K. Ali A. M. Ho W. Y. Alitheen N. B. Hamid M. (2012). scFv antibody: principles and clinical application. Clin. Dev. Immunol. 2012:980250. 10.1155/2012/980250 22474489 PMC3312285

[B3] Alejandra W. P. Miriam Irene J. P. Fabio Antonio G. S. Patricia R. R. Elizabeth T. A. Aleman-Aguilar J. P.. (2023). Production of monoclonal antibodies for therapeutic purposes: a review. Int. Immunopharmacol. 120:110376. 10.1016/j.intimp.2023.110376 37244118

[B4] Andreu-Sánchez S. Bourgonje A. R. Vogl T. Kurilshikov A. Leviatan S. Ruiz-Moreno A. J.. (2023). Phage display sequencing reveals that genetic, environmental, and intrinsic factors influence variation of human antibody epitope repertoire. Immunity 56, 1376-1392. 10.1016/j.immuni.2023.04.003 37164013 PMC12166656

[B5] Arrieta O. Zatarain-Barrón Z. L. Cardona A. F. Carmona A. Lopez-Mejia M. (2017). Ramucirumab in the treatment of non-small cell lung cancer. Expert Opin. Drug Saf. 16, 637–644. 10.1080/14740338.2017.1313226 28395526

[B6] Bandyopadhyay A. Das T. Nandy S. Sahib S. Preetam S. Gopalakrishnan A. V.. (2023). Ligand-based active targeting strategies for cancer theranostics. Naunyn. Schmiedebergs Arch. Pharmacol. 396, 3417–3441. 10.1007/s00210-023-02612-4 37466702

[B7] Bang Y. J. Ruiz E. Y. Van Cutsem E. Lee K. W. Wyrwicz L. Schenker M.. (2018). Phase III, randomised trial of avelumab versus physician's choice of chemotherapy as third-line treatment of patients with advanced gastric or gastro-oesophageal junction cancer: primary analysis of JAVELIN Gastric 300. Ann. Oncol. 29, 2052–2060. 10.1093/annonc/mdy264 30052729 PMC6225815

[B8] Bang Y. J. Van Cutsem E. Feyereislova A. Chung H. C. Shen L. Sawaki A.. (2010). Trastuzumab in combination with chemotherapy versus chemotherapy alone for treatment of HER2-positive advanced gastric or gastro-oesophageal junction cancer (ToGA): a phase 3, open-label, randomised controlled trial. Lancet 376, 687–697. 10.1016/S0140-6736(10)61121-X 20728210

[B9] Bathula N. V. Bommadevara H. Hayes J. M. (2021). Nanobodies: the future of antibody-based immune therapeutics. Cancer Biother. Radiopharm. 36, 109–122. 10.1089/cbr.2020.3941 32936001

[B10] Beghetto E. Gargano N. (2011). Lambda-display: a powerful tool for antigen discovery. Molecules 16, 3089–3105. 10.3390/molecules16043089 21490557 PMC6260602

[B11] Bichet M. C. Adderley J. Avellaneda-Franco L. Magnin-Bougma I. Torriero-Smith N. Gearing L. J.. (2023). Mammalian cells internalize bacteriophages and use them as a resource to enhance cellular growth and survival. PLoS Biol. 21:e3002341. 10.1371/journal.pbio.3002341 37883333 PMC10602308

[B12] Binnewies M. Roberts E. W. Kersten K. Chan V. Fearon D. F. Merad M.. (2018). Understanding the tumor immune microenvironment (TIME) for effective therapy. Nat. Med. 24, 541–550. 10.1038/s41591-018-0014-x 29686425 PMC5998822

[B13] Borek A. Sokolowska-Wedzina A. Chodaczek G. Otlewski J. (2018). Generation of high-affinity, internalizing anti-FGFR2 single-chain variable antibody fragment fused with Fc for targeting gastrointestinal cancers. PLoS ONE 13:e0192194. 10.1371/journal.pone.0192194 29420662 PMC5805272

[B14] Cancer Genome Atlas Research Network (2014). Comprehensive molecular characterization of gastric adenocarcinoma. Nature 513, 202–209. 10.1038/nature13480 25079317 PMC4170219

[B15] Casak S. J. Fashoyin-Aje I. Lemery S. J. Zhang L. Jin R. Li H.. (2015). FDA Approval Summary: Ramucirumab for Gastric Cancer. Clin. Cancer Res. 21, 3372–3376. 10.1158/1078-0432.CCR-15-0600 26048277

[B16] Chang C. Guo W. Yu X. Guo C. Zhou N. Guo X.. (2023). Engineered M13 phage as a novel therapeutic bionanomaterial for clinical applications: from tissue regeneration to cancer therapy. Mater. Today Bio. 20:100612. 10.1016/j.mtbio.2023.100612 37063776 PMC10102448

[B17] Chen P. C. Guo X. S. Zhang H. E. Dubey G. K. Geng Z. Z. Fierke C. A.. (2024). Leveraging a phage-encoded noncanonical amino acid: a novel pathway to potent and selective epigenetic reader protein inhibitors. ACS Cent. Sci. 10, 782–792. 10.1021/acscentsci.3c01419 38680566 PMC11046469

[B18] Chi Y. H. Hsiao J. K. Lin M. H. Chang C. Lan C. H. Wu H. C. (2017). Lung Cancer-targeting peptides with multi-subtype indication for combinational drug delivery and molecular imaging. Theranostics 7, 1612–1632. 10.7150/thno.17573 28529640 PMC5436516

[B19] Chongchai A. Waramit S. Suwan K. Al-Bahrani M. Udomruk S. Phitak T.. (2021). Bacteriophage-mediated therapy of chondrosarcoma by selective delivery of the tumor necrosis factor alpha (TNFα) gene. Faseb. J. 35:e21487. 10.1096/fj.202002539R 33811705

[B20] Conti C. B. Agnesi S. Scaravaglio M. Masseria P. Dinelli M. E. Oldani M.. (2023). Early gastric cancer: update on prevention, diagnosis and treatment. Int. J. Environ. Res. Public Health 20:2149. 10.3390/ijerph20032149 36767516 PMC9916026

[B21] Dedrick R. M. Freeman K. G. Nguyen J. A. Bahadirli-Talbott A. Smith B. E. Wu A. E.. (2021). Potent antibody-mediated neutralization limits bacteriophage treatment of a pulmonary *Mycobacterium abscessus* infection. Nat. Med. 27, 1357–1361. 10.1038/s41591-021-01403-9 34239133 PMC8571776

[B22] Deutscher S. L. (2010). Phage display in molecular imaging and diagnosis of cancer. Chem. Rev. 110, 3196–3211. 10.1021/cr900317f 20170129 PMC2868952

[B23] Duan T. Ferguson M. Yuan L. Xu F. Li G. (2009). Human monoclonal fab antibodies against west nile virus and its neutralizing activity analyzed *in Vitro and in Vivo*. J. Antivir. Antiretrovir. 1, 36–42. 10.4172/jaa.1000005 20505850 PMC2875541

[B24] Ecker D. M. Jones S. D. Levine H. L. (2015). The therapeutic monoclonal antibody market. MAbs 7, 9–14. 10.4161/19420862.2015.989042 25529996 PMC4622599

[B25] Esteva A. Kuprel B. Novoa R. A. Ko J. Swetter S. M. Blau H. M.. (2017). Dermatologist-level classification of skin cancer with deep neural networks. Nature 542, 115–118. 10.1038/nature21056 28117445 PMC8382232

[B26] Farrington G. K. Caram-Salas N. Haqqani A. S. Brunette E. Eldredge J. Pepinsky B.. (2014). A novel platform for engineering blood-brain barrier-crossing bispecific biologics. Faseb. J. 28, 4764–4778. 10.1096/fj.14-253369 25070367

[B27] Feng G. K. Liu R. B. Zhang M. Q. Ye X. X. Zhong Q. Xia Y. F.. (2014). SPECT and near-infrared fluorescence imaging of breast cancer with a neuropilin-1-targeting peptide. J. Control. Release. 192, 236–242. 10.1016/j.jconrel.2014.07.039 25058570

[B28] Fuchs C. S. Tomasek J. Yong C. J. Dumitru F. Passalacqua R. Goswami C.. (2014). Ramucirumab monotherapy for previously treated advanced gastric or gastro-oesophageal junction adenocarcinoma (REGARD): an international, randomised, multicentre, placebo-controlled, phase 3 trial. Lancet 383, 31–39. 10.1016/S0140-6736(13)61719-5 24094768

[B29] Garnock-Jones K. P. (2016). Necitumumab: first global approval. Drugs 76, 283–289. 10.1007/s40265-015-0537-0 26729188

[B30] Gillespie J. W. Wei L. Petrenko V. A. (2016). Selection of Lung Cancer-Specific Landscape Phage for Targeted Drug Delivery. Comb. Chem. High Throughput Screen 19, 412–422. 10.2174/1386207319666160420141024 27095536 PMC5066567

[B31] Hairul Bahara N. H. Tye G. J. Choong Y. S. Ong E. B. Ismail A. Lim T. S. (2013). Phage display antibodies for diagnostic applications. Biologicals 41, 209–216. 10.1016/j.biologicals.2013.04.001 23647952

[B32] Hajitou A. (2010). Targeted systemic gene therapy and molecular imaging of cancer contribution of the vascular-targeted AAVP vector. Adv. Genet. 69, 65–82. 10.1016/S0065-2660(10)69008-6 20807602

[B33] Hajitou A. Trepel M. Lilley C. E. Soghomonyan S. Alauddin M. M. Marini F. C. 3rd. (2006). A hybrid vector for ligand-directed tumor targeting and molecular imaging. Cell 125, 385–398. 10.1016/j.cell.2006.02.042 16630824

[B34] Harvey E. P. Shin J. E. Skiba M. A. Nemeth G. R. Hurley J. D. Wellner A.. (2022). An in silico method to assess antibody fragment polyreactivity. Nat. Commun. 13:7554. 10.1038/s41467-022-35276-4 36477674 PMC9729196

[B35] Heine P. A. Ballmann R. Thevarajah P. Russo G. Moreira G. Hust M. (2023). Biomarker Discovery by ORFeome Phage Display. Methods Mol. Biol. 2702, 543–561. 10.1007/978-1-0716-3381-6_27 37679638

[B36] Helms H. C. Abbott N. J. Burek M. Cecchelli R. Couraud P. O. Deli M. A.. (2016). *In vitro* models of the blood-brain barrier: An overview of commonly used brain endothelial cell culture models and guidelines for their use. J. Cereb. Blood Flow Metab. 36, 862–890. 10.1177/0271678X16630991 26868179 PMC4853841

[B37] Hood J. D. Bednarski M. Frausto R. Guccione S. Reisfeld R. A. Xiang R.. (2002). Tumor regression by targeted gene delivery to the neovasculature. Science 296, 2404–2407. 10.1126/science.1070200 12089446

[B38] Hosseinidoust Z. (2017). Phage-Mediated Gene Therapy. Curr. Gene Ther. 17, 120–126. 10.2174/1566523217666170510151940 28494733

[B39] Hu Y. Romão E. Vincke C. Brys L. Elkrim Y. Vandevenne M.. (2021). Intrabody targeting HIF-1α mediates transcriptional downregulation of target genes related to solid tumors. Int. J. Mol. Sci. 22:12335. 10.3390/ijms222212335 34830219 PMC8625554

[B40] Istomina P. V. Gorchakov A. A. Paoin C. Yamabhai M. (2024). Phage display for discovery of anticancer antibodies. N. Biotechnol. 83, 205–218. 10.1016/j.nbt.2024.08.506 39186973

[B41] Jin H. Gao X. Xiao L. He H. Cheng S. Zhang C.. (2021). Screening and identification of a specific peptide binding to breast cancer cells from a phage-displayed peptide library. Biotechnol. Lett. 43, 153–164. 10.1007/s10529-020-03044-3 33145670

[B42] Jończyk E. Kłak M. Miedzybrodzki R. Górski A. (2011). The influence of external factors on bacteriophages–review. Folia Microbiol. (Praha) 56, 191–200. 10.1007/s12223-011-0039-8 21625877 PMC3131515

[B43] Juds C. Schmidt J. Weller M. G. Lange T. Beck U. Conrad T.. (2020). Combining phage display and next-generation sequencing for materials sciences: a case study on probing polypropylene surfaces. J. Am. Chem. Soc. 142, 10624–10628. 10.1021/jacs.0c03482 32460497

[B44] Jun J. K. Choi K. S. Lee H. Y. Suh M. Park B. Song S. H.. (2017). Effectiveness of the Korean national cancer screening program in reducing gastric cancer mortality. Gastroenterology 152, 1319-1328. 10.1053/j.gastro.2017.01.029 28147224

[B45] Khan U. Shah M. A. (2019). Ramucirumab for the treatment of gastric or gastro-esophageal junction cancer. Expert Opin. Biol. Ther. 19, 1135–1141. 10.1080/14712598.2019.1656715 31452409

[B46] Kia A. Przystal J. M. Nianiaris N. Mazarakis N. D. Mintz P. J. Hajitou A. (2012). Dual systemic tumor targeting with ligand-directed phage and Grp78 promoter induces tumor regression. Mol. Cancer Ther. 11, 2566–2577. 10.1158/1535-7163.MCT-12-0587 23053496 PMC3521030

[B47] Körfer J. Lordick F. Hacker U. T. (2021). Molecular targets for gastric cancer treatment and future perspectives from a clinical and translational point of view. Cancers 13:5216. 10.3390/cancers13205216 34680363 PMC8533881

[B48] Kumar R. Parray H. A. Shrivastava T. Sinha S. Luthra K. (2019). Phage display antibody libraries: a robust approach for generation of recombinant human monoclonal antibodies. Int. J. Biol. Macromol. 135, 907–918. 10.1016/j.ijbiomac.2019.06.006 31170490

[B49] Kwak M. H. Yang S. M. Yun S. K. Kim S. Choi M. G. Park J. M. (2021). Identification and validation of LGR5-binding peptide for molecular imaging of gastric cancer. Biochem. Biophys. Res. Commun. 580, 93–99. 10.1016/j.bbrc.2021.09.073 34628260

[B50] Kwak M. H. Yun S. K. Yang S. M. Myeong S. Park J. M. (2024). Gastric cancer specific drug delivery with hydrophilic peptide probe conjugation. Biomater. Sci. 12, 440–452. 10.1039/D3BM01590D 38054470

[B51] LeCun Y. Bengio Y. Hinton G. (2015). Deep learning. Nature 521, 436–444. 10.1038/nature14539 26017442

[B52] Ledsgaard L. Ljungars A. Rimbault C. Sørensen C. V. Tulika T. Wade J.. (2022). Advances in antibody phage display technology. Drug Discov. Today 27, 2151–2169. 10.1016/j.drudis.2022.05.002 35550436

[B53] Lee M. Cho H. J. Park K. S. Jung H. Y. (2022). ELK3 Controls gastric cancer cell migration and invasion by regulating ECM remodeling-related genes. Int. J. Mol. Sci. 23:3709. 10.3390/ijms23073709 35409069 PMC8998440

[B54] Lehti T. A. Pajunen M. I. Skog M. S. Finne J. (2017). Internalization of a polysialic acid-binding *Escherichia coli* bacteriophage into eukaryotic neuroblastoma cells. Nat. Commun. 8:1915. 10.1038/s41467-017-02057-3 29203765 PMC5715158

[B55] Leung W. K. Wu M. S. Kakugawa Y. Kim J. J. Yeoh K. G. Goh K. L.. (2008). Screening for gastric cancer in Asia: current evidence and practice. Lancet Oncol. 9, 279–287. 10.1016/S1470-2045(08)70072-X 18308253

[B56] Li J. Lee A. S. (2006). Stress induction of GRP78/BiP and its role in cancer. Curr. Mol. Med. 6, 45–54. 10.2174/156652406775574523 16472112

[B57] Li X. Sun Z. Peng G. Xiao Y. Guo J. Wu B.. (2022). Single-cell RNA sequencing reveals a pro-invasive cancer-associated fibroblast subgroup associated with poor clinical outcomes in patients with gastric cancer. Theranostics 12, 620–638. 10.7150/thno.60540 34976204 PMC8692898

[B58] Liu J. Liu C. Ma Y. Pan X. Chu R. Yao S.. (2024). STING inhibitors sensitize platinum chemotherapy in ovarian cancer by inhibiting the CGAS-STING pathway in Cancer-Associated Fibroblasts (CAFs). Cancer Lett. 588:216700. 10.1016/j.canlet.2024.216700 38373690

[B59] Lohray R. Verma K. K. Wang L. L. Haynes D. Lewis D. J. (2023). Avelumab for advanced merkel cell carcinoma: global real-world data on patient response and survival. Pragmat. Obs. Res. 14, 149–154. 10.2147/POR.S398151 38021416 PMC10658947

[B60] Lu D. Jimenez X. Zhang H. Bohlen P. Witte L. Zhu Z. (2002). Selection of high affinity human neutralizing antibodies to VEGFR2 from a large antibody phage display library for antiangiogenesis therapy. Int. J. Cancer 97, 393–399. 10.1002/ijc.1634 11774295

[B61] Lu D. Shen J. Vil M. D. Zhang H. Jimenez X. Bohlen P.. (2003). Tailoring *in vitro* selection for a picomolar affinity human antibody directed against vascular endothelial growth factor receptor 2 for enhanced neutralizing activity. J. Biol. Chem. 278, 43496–43507. 10.1074/jbc.M307742200 12917408

[B62] Macedo F. Ladeira K. Longatto-Filho A. Martins S. F. (2017). Gastric cancer and angiogenesis: is VEGF a useful biomarker to assess progression and remission. JGC 17, 1–10. 10.5230/jgc.2017.17.e1 28337358 PMC5362829

[B63] Mahdavi S. Z. B. Oroojalian F. Eyvazi S. Hejazi M. Baradaran B. Pouladi N.. (2022). An overview on display systems (phage, bacterial, and yeast display) for production of anticancer antibodies; advantages and disadvantages. Int. J. Biol. Macromol. 208, 421–442. 10.1016/j.ijbiomac.2022.03.113 35339499

[B64] Malm M. Frejd F. Y. Ståhl S. Löfblom J. (2016). Targeting HER3 using mono- and bispecific antibodies or alternative scaffolds. MAbs 8, 1195–1209. 10.1080/19420862.2016.1212147 27532938 PMC5058629

[B65] Mishra R. Patel H. Alanazi S. Yuan L. Garrett J. T. (2018). HER3 signaling and targeted therapy in cancer. Oncol. Rev. 12:355. 10.4081/oncol.2018.355 30057690 PMC6047885

[B66] Møller-Olsen C. Ho S. F. S. Shukla R. D. Feher T. Sagona A. P. (2018). Engineered K1F bacteriophages kill intracellular *Escherichia coli* K1 in human epithelial cells. Sci. Rep. 8:17559. 10.1038/s41598-018-35859-6 30510202 PMC6277420

[B67] Muruganandam A. Tanha J. Narang S. Stanimirovic D. (2002). Selection of phage-displayed llama single-domain antibodies that transmigrate across human blood-brain barrier endothelium. Faseb. J. 16, 240–242. 10.1096/fj.01-0343fje 11772942

[B68] Newman M. R. Benoit D. S. W. (2018). *In Vivo* translation of peptide-targeted drug delivery systems discovered by phage display. Bioconjug. Chem. 29, 2161–2169. 10.1021/acs.bioconjchem.8b00285 29889510 PMC6543811

[B69] Nicastro J. Sheldon K. Slavcev R. A. (2014). Bacteriophage lambda display systems: developments and applications. Appl. Microbiol. Biotechnol. 98, 2853–2866. 10.1007/s00253-014-5521-1 24442507

[B70] Ooi V. Y. Yeh T. Y. (2024). Recent advances and mechanisms of phage-based therapies in cancer treatment. Int. J. Mol. Sci. 25:9938. 10.3390/ijms25189938 39337427 PMC11432602

[B71] Paoloni M. C. Tandle A. Mazcko C. Hanna E. Kachala S. Leblanc A.. (2009). Launching a novel preclinical infrastructure: comparative oncology trials consortium directed therapeutic targeting of TNFalpha to cancer vasculature. PLoS ONE 4:e4972. 10.1371/journal.pone.0004972 19330034 PMC2659423

[B72] Peng Y. Prater A. R. Deutscher S. L. (2017). Targeting aggressive prostate cancer-associated CD44v6 using phage display selected peptides. Oncotarget 8, 86747–86768. 10.18632/oncotarget.21421 29156833 PMC5689723

[B73] Piggott A. M. Karuso P. (2016). Identifying the cellular targets of natural products using T7 phage display. Nat. Prod. Rep. 33, 626–636. 10.1039/C5NP00128E 26964751

[B74] Poplin R. Chang P. C. Alexander D. Schwartz S. Colthurst T. Ku A.. (2018). A universal SNP and small-indel variant caller using deep neural networks. Nat. Biotechnol. 36, 983–987. 10.1038/nbt.4235 30247488

[B75] Przystal J. M. Waramit S. Pranjol M. Z. I. Yan W. Chu G. Chongchai A.. (2019). Efficacy of systemic temozolomide-activated phage-targeted gene therapy in human glioblastoma. EMBO Mol. Med. 11:8492. 10.15252/emmm.201708492 30808679 PMC6460351

[B76] Qi H. Ma M. Lai D. Tao S. C. (2021). Phage display: an ideal platform for coupling protein to nucleic acid. Acta Biochim. Biophys. Sin. 53, 389–399. 10.1093/abbs/gmab006 33537750

[B77] Rahim M. A. Jan N. Khan S. Shah H. Madni A. Khan A.. (2021). Recent advancements in stimuli responsive drug delivery platforms for active and passive cancer targeting. Cancers 13:670. 10.3390/cancers13040670 33562376 PMC7914759

[B78] Ren Z. Black L. W. (1998). Phage T4 SOC and HOC display of biologically active, full-length proteins on the viral capsid. Gene 215, 439–444. 10.1016/S0378-1119(98)00298-4 9714843

[B79] Reynolds K. L. Bedard P. L. Lee S. H. Lin C. C. Tabernero J. Alsina M.. (2017). A phase I open-label dose-escalation study of the anti-HER3 monoclonal antibody LJM716 in patients with advanced squamous cell carcinoma of the esophagus or head and neck and HER2-overexpressing breast or gastric cancer. BMC Cancer 17:646. 10.1186/s12885-017-3641-6 28899363 PMC5596462

[B80] Rossez Y. Burtea C. Laurent S. Gosset P. Léonard R. Gonzalez W.. (2016). Early detection of colonic dysplasia by magnetic resonance molecular imaging with a contrast agent raised against the colon cancer marker MUC5AC. Contrast. Media Mol. Imag. 11, 211–221. 10.1002/cmmi.1682 26762591

[B81] Sales-Dias J. Ferreira A. Lamy M. Domenici G. Monteiro S. M. S. Pires A.. (2021). Development of antibodies against the notch ligand Delta-Like-1 by phage display with activity against breast cancer cells. N. Biotechnol. 64, 17–26. 10.1016/j.nbt.2021.05.003 33992842

[B82] Sanmukh S. G. Dos Santos N. J. Barquilha C. N. Cucielo M. S. de Carvalho M. Dos Reis P. P.. (2021a). Bacteriophages M13 and T4 increase the expression of anchorage-dependent survival pathway genes and down regulate androgen receptor expression in LNCaP prostate cell line. Viruses 13:1754. 10.3390/v13091754 34578333 PMC8473360

[B83] Sanmukh S. G. Dos Santos N. J. Nascimento Barquilha C. De Carvalho M. Pintor Dos Reis P. Delella F. K.. (2023). Bacterial RNA virus MS2 exposure increases the expression of cancer progression genes in the LNCaP prostate cancer cell line. Oncol Lett. 25:86. 10.3892/ol.2023.13672 36760518 PMC9878357

[B84] Sanmukh S. G. Felisbino S. L. (2018). Development of pipette tip gap closure migration assay (s-ARU method) for studying semi-adherent cell lines. Cytotechnology 70, 1685–1695. 10.1007/s10616-018-0245-1 30069611 PMC6269371

[B85] Sanmukh S. G. Santos N. J. Barquilha C. N. Dos Santos S. A. A. Duran B. O. S. Delella F. K.. (2021b). Exposure to bacteriophages T4 and M13 increases integrin gene expression and impairs migration of human PC-3 prostate cancer cells. Antibiotics 10:1202. 10.3390/antibiotics10101202 34680783 PMC8532711

[B86] Saw P. E. Song E. W. (2019). Phage display screening of therapeutic peptide for cancer targeting and therapy. Protein Cell. 10, 787–807. 10.1007/s13238-019-0639-7 31140150 PMC6834755

[B87] Shen W. Shi P. Dong Q. Zhou X. Chen C. Sui X.. (2023). Discovery of a novel dual-targeting D-peptide to block CD24/Siglec-10 and PD-1/PD-L1 interaction and synergize with radiotherapy for cancer immunotherapy. J. Immunother. Cancer 11:e007068. 10.1136/jitc-2023-007068 37344099 PMC10314633

[B88] Shim H. (2016). Therapeutic Antibodies by Phage Display. Curr. Pharm. Des. 22, 6538–6559. 10.2174/1381612822666160923113714 27669967

[B89] Shivachandra S. B. Li Q. Peachman K. K. Matyas G. R. Leppla S. H. Alving C. R.. (2007). Multicomponent anthrax toxin display and delivery using bacteriophage T4. Vaccine 25, 1225–1235. 10.1016/j.vaccine.2006.10.010 17069938 PMC1888565

[B90] Smith T. L. Yuan Z. Cardó-Vila M. Sanchez Claros C. Adem A. Cui M. H.. (2016). AAVP displaying octreotide for ligand-directed therapeutic transgene delivery in neuroendocrine tumors of the pancreas. Proc. Nat. Acad. Sci. U. S. A. 113, 2466–2471. 10.1073/pnas.1525709113 26884209 PMC4780640

[B91] Stutz C. C. Georgieva J. V. Shusta E. V. (2018). Coupling brain perfusion screens and next generation sequencing to identify blood-brain barrier binding antibodies. AIChE J. 64, 4229–4236. 10.1002/aic.16360 30872841 PMC6411078

[B92] Sui Y. Zhu R. Hu W. Zhang W. Zhu H. Gong M.. (2021). Phage display screening identifies a prostate specific antigen (PSA)(-/lo) prostate cancer cell specific peptide to retard castration resistance of prostate cancer. Transl. Oncol. 14:101020. 10.1016/j.tranon.2021.101020 33508757 PMC7844130

[B93] Sun Y. Ban B. Bradbury A. Ansari G. A. Blake D. A. (2016). Combining yeast display and competitive facs to select rare hapten-specific clones from recombinant antibody libraries. Anal. Chem. 88, 9181–9189. 10.1021/acs.analchem.6b02334 27571429 PMC5032104

[B94] Suwan K. Yata T. Waramit S. Przystal J. M. Stoneham C. A. Bentayebi K.. (2019). Next-generation of targeted AAVP vectors for systemic transgene delivery against cancer. Proc. Nat. Acad. Sci. U. S. A. 116, 18571–18577. 10.1073/pnas.1906653116 31375630 PMC6744886

[B95] Sweere J. M. Van Belleghem J. D. Ishak H. Bach M. S. Popescu M. Sunkari V.. (2019). Bacteriophage trigger antiviral immunity and prevent clearance of bacterial infection. Science 363:9691. 10.1126/science.aat9691 30923196 PMC6656896

[B96] Takahashi S. Kobayashi T. Tomomatsu J. Ito Y. Oda H. Kajitani T.. (2017). LJM716 in Japanese patients with head and neck squamous cell carcinoma or HER2-overexpressing breast or gastric cancer. Cancer Chemother. Pharmacol. 79, 131–138. 10.1007/s00280-016-3214-4 27942917 PMC5225197

[B97] Tandle A. Hanna E. Lorang D. Hajitou A. Moya C. A. Pasqualini R.. (2009). Tumor vasculature-targeted delivery of tumor necrosis factor-alpha. Cancer 115, 128–139. 10.1002/cncr.24001 19090007 PMC8385542

[B98] Tao P. Zhu J. Mahalingam M. Batra H. Rao V. B. (2019). Bacteriophage T4 nanoparticles for vaccine delivery against infectious diseases. Adv. Drug Deliv. Rev. 145, 57–72. 10.1016/j.addr.2018.06.025 29981801 PMC6759415

[B99] Timur S. S. Gürsoy R. N. (2021). The role of peptide-based therapeutics in oncotherapy. J. Drug Target. 29, 1048–1062. 10.1080/1061186X.2021.1906884 33775190

[B100] Trepel M. Stoneham C. A. Eleftherohorinou H. Mazarakis N. D. Pasqualini R. Arap W.. (2009). A heterotypic bystander effect for tumor cell killing after adeno-associated virus/phage-mediated, vascular-targeted suicide gene transfer. Mol. Cancer Ther. 8, 2383–2391. 10.1158/1535-7163.MCT-09-0110 19671758 PMC2871293

[B101] Trott M. Wei,β S. Antoni S. Koch J. von Briesen H. Hust M.. (2014). Functional characterization of two scFv-Fc antibodies from an HIV controller selected on soluble HIV-1 env complexes: a neutralizing V3- and a trimer-specific gp41 antibody. PLoS ONE 9:e97478. 10.1371/journal.pone.0097478 24828352 PMC4020869

[B102] Vascon F. Gasparotto M. Giacomello M. Cendron L. Bergantino E. Filippini F.. (2020). Protein electrostatics: From computational and structural analysis to discovery of functional fingerprints and biotechnological design. Comput. Struct. Biotechnol. J. 18, 1774–1789. 10.1016/j.csbj.2020.06.029 32695270 PMC7355722

[B103] Waehler R. Russell S. J. Curiel D. T. (2007). Engineering targeted viral vectors for gene therapy. Nat. Rev. Genet. 8, 573–587. 10.1038/nrg2141 17607305 PMC7097627

[B104] Wainberg M. Merico D. Delong A. Frey B. J. (2018). Deep learning in biomedicine. Nat. Biotechnol. 36, 829–838. 10.1038/nbt.4233 30188539

[B105] Wang J. Liu Y. Teesalu T. Sugahara K. N. Kotamrajua V. R. Adams J. D.. (2011). Selection of phage-displayed peptides on live adherent cells in microfluidic channels. Proc. Nat. Acad. Sci. U. S. A. 108, 6909–6914. 10.1073/pnas.1014753108 21486998 PMC3084056

[B106] Wang J. Tan Y. Ling J. Zhang M. Li L. Liu W.. (2021a). Highly paralleled emulsion droplets for efficient isolation, amplification, and screening of cancer biomarker binding phages. Lab. Chip. 21, 1175–1184. 10.1039/D0LC01146K 33554995

[B107] Wang J. Xiu J. Baca Y. Battaglin F. Arai H. Kawanishi N.. (2021b). Large-scale analysis of KMT2 mutations defines a distinctive molecular subset with treatment implication in gastric cancer. Oncogene 40, 4894–4905. 10.1038/s41388-021-01840-3 34163031

[B108] Wang W. Chen X. Li T. Li Y. Wang R. He D.. (2013). Screening a phage display library for a novel FGF8b-binding peptide with anti-tumor effect on prostate cancer. Exp. Cell. Res. 319, 1156–1164. 10.1016/j.yexcr.2013.02.007 23466786

[B109] Wang Y. Chen Y. L. Xu H. Rana G. E. Tan X. He M.. (2024). Comparison of “framework Shuffling” and “CDR Grafting” in humanization of a PD-1 murine antibody. Front. Immunol. 15:1395854. 10.3389/fimmu.2024.1395854 39076979 PMC11284016

[B110] Wang Z. Zhao C. Ding J. Chen Y. Liu J. Hou X.. (2023). Screening, construction, and preliminary evaluation of cldn18.2-specific peptides for noninvasive molecular imaging. ACS Pharmacol. Transl. Sci. 6, 1829–1840. 10.1021/acsptsci.3c00165 38093841 PMC10714438

[B111] Wu Y. Zhu M. Sun B. Chen Y. Huang Y. Gai J.. (2024). A humanized trivalent Nectin-4-targeting nanobody drug conjugate displays potent antitumor activity in gastric cancer. J. Nanobiotechnol. 22:256. 10.1186/s12951-024-02521-5 38755613 PMC11097425

[B112] Xia J. Bi H. Yao Q. Qu S. Zong Y. (2006). Construction of human ScFv phage display library against ovarian tumor. J. Huazhong Univ. Sci. Technol. Med. Sci. 26, 497–499. 10.1007/s11596-006-0502-y 17219950

[B113] Xiang Y. Sang Z. Bitton L. Xu J. Liu Y. Schneidman-Duhovny D.. (2021). Integrative proteomics identifies thousands of distinct, multi-epitope, and high-affinity nanobodies. Cell. Syst. 12, 220-234. 10.1016/j.cels.2021.01.003 33592195 PMC7979497

[B114] Yang Zhou J. Suwan K. Hajitou A. (2020). Initial steps for the development of a phage-mediated gene replacement therapy using CRISPR-Cas9 technology. J. Clin. Med. 9:1498. 10.3390/jcm9051498 32429407 PMC7290871

[B115] Yata T. Lee E. L. Suwan K. Syed N. Asavarut P. Hajitou A. (2015). Modulation of extracellular matrix in cancer is associated with enhanced tumor cell targeting by bacteriophage vectors. Mol. Cancer 14:110. 10.1186/s12943-015-0383-4 26037383 PMC4451735

[B116] Yu J. Zhang S. Zhao B. (2016). Differences and correlation of serum CEA, CA19-9 and CA72-4 in gastric cancer. Mol. Clin. Oncol. 4, 441–449. 10.3892/mco.2015.712 26998301 PMC4774443

[B117] Yu T. Sun Z. Cao X. Pang Q. Deng H. (2022). Recent trends in T7 phage application in diagnosis and treatment of various diseases. Int. Immunopharmacol. 110:109071. 10.1016/j.intimp.2022.109071 35978521

[B118] Yue H. Shan L. Bin L. (2018). The significance of OLGA and OLGIM staging systems in the risk assessment of gastric cancer: a systematic review and meta-analysis. Gastric Cancer 21, 579–587. 10.1007/s10120-018-0812-3 29460004

[B119] Żaczek M. Łusiak-Szelachowska M. Jończyk-Matysiak E. Weber-Dabrowska B. Miedzybrodzki R. Owczarek B.. (2016). Antibody production in response to Staphylococcal MS-1 Phage cocktail in patients undergoing phage therapy. Front. Microbiol. 7:1681. 10.3389/fmicb.2016.01681 27822205 PMC5075762

[B120] Zalewska-Piatek B. (2023). Phage Therapy-Challenges, Opportunities and Future Prospects. Pharmaceuticals 16:1638. 10.3390/ph16121638 38139765 PMC10747886

[B121] Zarei B. Javidan Z. Fatemi E. Rahimi Jamnani F. Khatami S. Khalaj V. (2020). Targeting c-Met on gastric cancer cells through a fully human fab antibody isolated from a large naive phage antibody library. Daru 28, 221–235. 10.1007/s40199-020-00334-z 32193747 PMC7238820

[B122] Zhang D. Huang J. Li W. Zhang Z. Zhu M. Feng Y.. (2020). Screening and identification of a CD44v6 specific peptide using improved phage display for gastric cancer targeting. Ann. Transl. Med. 8:1442. 10.21037/atm-19-4781 33313187 PMC7723568

[B123] Zhang D. Jia H. Wang Y. Li W. M. Hou Y. C. Yin S. W.. (2015). A CD44 specific peptide developed by phage display for targeting gastric cancer. Biotechnol. Lett. 37, 2311–2320. 10.1007/s10529-015-1896-z 26140900

[B124] Zhang J. Zhang W. Yang M. Zhu W. Li M. Liang A.. (2021). Passive cancer targeting with a viral nanoparticle depends on the stage of tumorigenesis. Nanoscale 13, 11334–11342. 10.1039/D1NR01619A 34165123

[B125] Zhang L. Sun L. Wei R. Gao Q. He T. Xu C.. (2017). Intracellular *Staphylococcus aureus* control by virulent bacteriophages within MAC-T bovine mammary epithelial cells. Antimicrob. Agents Chemother. 61, e01990-16. 10.1128/AAC.01990-16 27919889 PMC5278684

[B126] Zhang T. Li X. He Y. Wang Y. Shen J. Wang S.. (2022a). Cancer-associated fibroblasts-derived HAPLN1 promotes tumour invasion through extracellular matrix remodeling in gastric cancer. Gastric Cancer 25, 346–359. 10.1007/s10120-021-01259-5 34724589 PMC8882084

[B127] Zhang W. J. Sui Y. X. Budha A. Zheng J. B. Sun X. J. Hou Y. C.. (2012). Affinity peptide developed by phage display selection for targeting gastric cancer. World J. Gastroenterol. 18, 2053–2060. 10.3748/wjg.v18.i17.2053 22563192 PMC3342603

[B128] Zhang Y. (2023). Evolution of phage display libraries for therapeutic antibody discovery. MAbs 15:2213793. 10.1080/19420862.2023.2213793 37222232 PMC10210849

[B129] Zhang Y. T. Wang S. H. Zhao L. Wang H. M. Wang L. Shi R. R.. (2024). Screening and identification of vascular endothelial cell targeting peptide in gastric cancer through novel integrated in vitro and in vivo strategy. BMC Cancer 24:1595. 10.1186/s12885-024-13375-3 39736630 PMC11687070

[B130] Zhang Z. Cui P. Zhu W. (2022b). Deep learning on graphs: a survey. IEEE Trans. Knowl. Data Eng. 34, 249–270. 10.1109/TKDE.2020.2981333

[B131] Zhao H. Nie D. Hu Y. Chen Z. Hou Z. Li M.. (2023a). Phage display-derived peptides and antibodies for bacterial infectious diseases therapy and diagnosis. Molecules 28:2621. 10.3390/molecules28062621 36985593 PMC10052323

[B132] Zhao Y. Lu X. Huang H. Yao Y. Liu H. Sun Y. (2023b). Dendrobium officinale polysaccharide converts M2 into M1 Subtype macrophage polarization via the STAT6/PPAR-r and JAGGED1/NOTCH1 signaling pathways to inhibit gastric cancer. Molecules 28:7062. 10.3390/molecules28207062 37894541 PMC10609635

[B133] Zhu Y. Zhu X. Wei X. Tang C. Zhang W. (2021). HER2-targeted therapies in gastric cancer. Biochim. Biophys. Acta Rev. Cancer 1876:188549. 10.1016/j.bbcan.2021.188549 33894300

[B134] Zou J. Glinsky V. V. Landon L. A. Matthews L. Deutscher S. L. (2005). Peptides specific to the galectin-3 carbohydrate recognition domain inhibit metastasis-associated cancer cell adhesion. Carcinogenesis 26, 309–318. 10.1093/carcin/bgh329 15528216

[B135] Zou J. Huss M. Abid A. Mohammadi P. Torkamani A. Telenti A. (2019). A primer on deep learning in genomics. Nat. Genet. 51, 12–18. 10.1038/s41588-018-0295-5 30478442 PMC11180539

